# Foreign Medico-Psychological Literature

**Published:** 1863-07

**Authors:** 


### FOREIGN MEDICO-PSYCHOLOGICAL LITERATURE.

OUR Retrospect of current Foreign Medico-Psychological Literature embraces the following subjects:—

1. The Colonization of the Insane.
2. Suicide in Bavaria.
3. On Muscular Hallucinations.

### 1.—*Discussion on the Colonization of the Insane.*—In the *Parisian Medico-Psychological Society.*

M. GIRARD DE CAILLEUX, in opening the discussion, contended that the study of insanity, and the treatment suitable to it, depends now, as formerly, upon two kinds of indications which it was necessary to fulfil. That these indications were physical and moral, and that the best treatment pointed out by science certainly consists in the individual treatment of the insane, but that the necessity for economy rendered it necessary to recur to the construction of asylums. Scientific investigation has brought out the utility of making a distinction between the insane who are able to live together, those whose violence affects others injuriously, and those whose excessive impressionability is governed by eccentricities and impulses which they are unable to resist. Hence arises a new order of indications—that of isolating these two last classes from patients under the most favourable conditions for treatment. These again should be placed not amongst men uncultivated and interested in their employment, but in a family where education and instruction have elevated the character and developed the intelligence. It is in this way that we have thought it would be advantageous to scatter the insane in cottages, under the direction of a physician who would be content to live an isolated life, and devote himself, notwithstanding the small remuneration, to the treatment of mental disease. There would also require to be associated with him some simple laborious and industrious families under his direction.

*M. Moreau* (de Tours) on rising, after some preliminary remarks, pointed out that it was not the radical insufficiency of asylum accommodation which originated the idea of colonization, but a profound examination of the scientific principles realized at Gheel.

Besides, by colonization he meant the system of a pure colony as carried out in Belgium, and not that kind of compromise which he had no repugnance to accept, though only as a kind of transition between the old and new system. And here he would inquire to what reduces itself all the exigencies required by the position of the insane? It is to heal him if he is curable, and if he is incurable to place him in the most advantageous hygienic conditions, and to create for him, so far as it is consistent with his own safety and that of others, a life similar to that he enjoyed in health. The first of these necessities might be accomplished in old asylums; but we cannot carry out the second, for just as much as the atmosphere, speaking physically and morally, in our great asylums is unfavourable, so much the more pure and vivifying is it in the bosom of a family in which the patient finds all those incessant cares, attention, and counsel, in a word, all that M. le Dr. Bulcken so justly denominates, the *patronage familial*. And in regard to *work*, in his opinion it is only in a colony we can derive all the advantages from it; for when the disease is acute, there is for each patient not only an attendant, but a companion, who, in watching over him, executes the same labour as himself. This, however, cannot be the case where there are six hundred patients; and during the twenty years M. Moreau spent at Bicêtre, he observed when the disease was of recent date there was a repugnance to labour, whilst the chronic insane could not be got to join in it. But in the colonial system these difficulties disappear, for the patient is a member of the family, and can be got to take part in the work, by which he accumulates a small purse which he sets apart for his own necessities. There is thus a removal from the monotony of an hospital where patients are subjected to occupations they feel they have no taste for; and besides, in a colony there are greater sources of amusement, one of the most active agents in cure, and amongst which we may enumerate the visits of friends, family fêtes, going to fairs, markets, &c. But it has been thought in a colony medical treatment is impossible, from the difficulty of visiting the patients so frequently as they ought to be. This objection, however, is only specious, for the curable can never be so numerous but they may be seen when necessary. The second class, those who have a small chance of cure, may be placed in a more eccentric zone, but still within reach of the medical staff; whilst the third class, those who require to be watched but not treated, may be placed at a greater distance, but never too far to prevent being visited once or twice, or oftener, each week. Matters being thus arranged, the colonial system appears exempt from inconvenience, when we add that the nurse, in cases of acute disease, sends the patient to the infirmary or central asylum. This infirmary, which all who have visited Gheel have recognised the necessity of, is destined to receive temporarily the recent arrivals in the colony, and those suffering from acute mania and recurrent attacks of insanity. It is, as it were, the completion of the system, for which he did not fear to express his warmest sympathies. As to the establishment of the colonial system in France it appears perfectly possible; and though the objection of the difference of character in the nation has been raised, this does not seem to require a serious refutation; for what time, chance, vitality have done in a neighbouring country, science and the will of enlightened men may be able to accomplish in this; and he persisted in believing that the creation in any country whatever of an establishment like that of Belgium would not meet with any serious obstacles.

*M. Billod*, in bearing testimony to the exactness of M. Falret's report, said, that if he had passed through Gheel by chance, he would not have been aware of its peculiar characteristic, though he was struck with its mournfully silent aspect, which could not render the residence of the insane in it more pleasant than in an asylum. The reason of this further investigation seemed explained to him. The insane, in fact, do not enjoy the unlimited liberty represented; the nurses, feeling not only their own responsibility, but that of the entire colony, leave the patients to themselves anything but willingly; whilst kept at home by their occupations, they cannot take their charges to walk, which is all the more necessary as they do not live in the most favourable hygienic conditions. The patients thus become sedentary in their habits, and the order which reigns in the colony depends upon conditions more or less restrictive, and these conditions, combined with the infirmary, approximates its organization to that of an asylum, constituting, if not an abandonment of the colonial system, at least a concession to the opposite one.

The adoption of the "patronage familial" system, he believed to be impossible, for two reasons:—first, because in so sceptical and positive an age we want the requisite amount of faith, its fundamental condition; and second, from the apprehension the insane inspire, they are generally surrounded by distrust, and continue to be an object of fear. This last objection, in fact, is not a conjectural one, for an attempt was made to establish this system in another part of Belgium, and was almost immediately given up on account of the resistance of the authorities to it. From this double impossibility of applying the system of Gheel it is useless to discuss its advantages and disadvantages; in any case it is only applicable to the chronic insane, but for these he would prefer entrusting them to their own families, an annual amount being paid to them by the state; and for want of a more honourable motive he would thus secure a guarantee of their care and solicitude for the insane in the interest they would have in the continuance of this allowance. The superintendent of the asylum ought to be judge of the cases which might be tried in this way, and the departmental inspector of foundlings ought to inspect those families who receive allowances for the maintenance of the insane.

*M. Baillarger* was struck with the number of cases of seclusion at Gheel, which seemed to be more liable to abuse than that in an asylum under the surveillance of a physician.

*M. Moreau* (de Tours) replied to some of the objections of MM. Billod and Baillarger, and stated that a colony could only be formed gradually, and by entrusting an insane workman whom they found of use to two or three families. They would in this way be encouraged to ask for more, and little by little a colony would be founded. At least an attempt was reasonable to develop a system which would include nearly three-fourths of the insane.

*M. Baillarger* reiterated the statement that seclusion was safer in an asylum, and mentioned that only eight years ago a foreign physician saw a patient at Gheel loaded with chains.

*M. Brierre de Boismont* argued from criminal statistics which he quoted that it would be imprudent to leave insane and hysterical women at large in the midst of villagers.

*M. J. Falret* stated that at Gheel the strait-jacket, hand-cuffs, &c., were still used, and that he had seen a woman wearing a strait-jacket, because the family were employed in labour she could not engage in; sixteen patients in 800 wore handcuffs and strait-jackets.

*M. Delasiaure*—In well conducted asylums only one patient in a hundred is under restraint.

*M. Baillarger*—Gheel may be suitable for the chronic insane, and those capable of engaging in work, but it is absurd to think of sending there those suffering from acute mental disease.

*M. Moreau* (de Tours) remarked that now since there is an infirmary, cases might be kept there until the acute stage were past.

*M. Girard de Cailleux*—In wishing to found agricultural colonies there has not been sufficiently taken into account the physiological conditions and new aptitudes produced in the insane by surrounding circumstances. Statistical observation proves that the sudden transition from a sedentary to an active life in the open air produces fatal results, and M. Billod has brought out this well in demanding the erection of workshops in accordance with the acquired habits of the patients. *(Annales Medico-Psychologiques.)*

---

### 2.—*Suicide in Bavaria.*

M. Majee in his work on this subject states, among other motives for suicide, that the price of food has great influence upon it, especially during the last few years since food has so greatly increased in price.

Suicides increase in proportion to the increase of the population, but in times of great political agitation the number of suicides diminishes, increasing again when quiet and order are re-established, doubtless the result of hopes deceived.

Suicide, he remarks, is also more frequent in towns than in the country, but that may be accounted for by the greater amount of population in the former.

Sex exercises an influence on this malady, as men are attacked in greater numbers than women, the proportion being 4 to 1; now as this difference relative to sex is not found in mental affections, although in the number of crimes, suicide can be but rarely attributed to a derangement of the intellectual faculties.

Violent deaths by suicide, by assassination, or by accident, taken as a whole, are three times more frequent among men than among women, and suicides by women are of more constant occurrence in towns than in the country. The greatest number of people commit suicide when arrived at manhood. In Bavaria the maximum occur between the ages of forty and fifty; under forty years of age and over sixty, there are more women in proportion; whilst between forty and sixty there are more men who commit this crime.

In a given number of people suicide is found to be three times more general among the Protestants than the Catholics, and about a third more frequent than among the Jews. In mixed provinces the frequency is in an inverse ratio to the number of the Catholic inhabitants. By way of compensation, crimes are more universal among these latter.

In agricultural populations this crime is nearly four times as rare as it is in industrial populations, and in years of dearness the proportion seems to increase in the towns more than in the country. Suicide, at least in Bavaria, is rather more frequent among married people; crime on the contrary is always more constantly committed by the unmarried. About half of the number who commit suicide enjoy good health; intellectual derangement has been satisfactorily found to exist in about a fifth, and bodily affections in about a fourth.

The greater number of patients were little favoured by family or fortune; but in about two-fifths their position and circumstances left nothing to be desired. Suicides from mental causes are more common among Catholics than Protestants.

Death by hanging is the method selected by half those who commit suicide in Bavaria and Germany; then, drowning by about a fourth. Ladies generally choose this latter method.

The greatest number of suicidal deaths occur in June, July, and August; the smaller number in the cold months of November, December, and January.

We see again in these facts how much the crime of suicide is influenced by national customs and modes of life. The Bavarians follow the English in that hanging is with them the most popular form of self-destruction. (*Social Science Review*).

---

### 3.—*On Muscular Hallucinations.* By M. Eugene Semerie.

Musculation,* or the sense of muscular activity, makes us conscious of our muscular efforts, and of the fatigue which results from them. Thus conceived, musculation should be regarded as having an existence distinct from touch, and I do not doubt that it will soon obtain a place in the classical treatises on physiology. The reasons in favour of this distinction long ago attracted the attention

* The word *musculation* was first used by Gerdy, who gave it quite a different meaning. I think it was Auguste Comte who first employed this word to denote the sense of muscular activity.

of some philosophers. Aristotle even, in his *Treatise on the Soul*, made the remark that, while the other senses could but reckon one single opposition by contraries, that of touch possessed several; hot and cold, dry and moist, hard and soft, besides many others of the same kind, and that we did not know clearly what was the unique characteristic of the sense of touch.

Cardan, that strange and subtle genius, who was able to study from his own experience the subject of tactile hallucinations, recognised the existence of a special sense giving consciousness of weight and its opposite. But it is only at a much more recent epoch that study has resulted in anything approaching the analysis of tactile sensations. Charles Bell in the first instance, and afterwards, almost in our own day, Belfield, Lefevre, Gerdy, MM. Beau and Landry, have been the promoters of this movement. The latter has summed up in an excellent work his opinions on this subject.

According to Auguste Comte, Blainville has pointed out in a very precise manner the existence of musculation. The following is what this great philosopher says on this point. "I believe we ought finally to acknowledge eight (senses) really distinct: one general, touch, and seven special; musculation, gustation, calorition, olfaction, audition, vision, and electrition. I rank the latter after Gall and Blainville, following their increasing speciality conformably with that of corresponding phenomena, and measured by successive appearance, in the animal scale. The two extremes alone require a special explanation. *With regard to the first, I adopt essentially the opinion of Blainville, who separates it from the general sense of pressure, reserving for it the direct appreciation of muscular efforts, and of the fatigue which they produce.* As to the last, its commonly slight development in man should not hinder the acknowledgment of its distinct existence, which is very decided in certain animals, and more or less common to all vertebrates." I believe we should search vainly in what remains to us of Blainville for so precise an opinion.

However this may be, musculation has, at the present day, acquired a right to be cited, and it is not my business to develope here all the reasons which concur in making it a special sense. These reasons have been largely dwelt upon by the authors whom I have quoted above, and M. Audiffard, in his inaugural thesis (Montpellier, 1859), recently furnished others, and made an ingenious application of them, attributing rheumatism to exaggerated sensations of calorition and musculation, that is to say, to a true neurosis of these two senses, of which the fluxionary state would in more than one case be only the consequence. "When we consider," says he, "that all fatigue makes itself felt more especially in the articulations, and in the muscular fascines, one would be almost tempted to see in that a confirmation of our supposition." The object which I propose is only to complete the history of this new sense in examining what happens to it in insanity, and to what order of hallucinations it gives rise.

One ought to presume the existence of muscular hallucinations, and even that they are frequent, for they have for their seat the vast muscular apparatus and its corresponding portions of the brain. I will cite in the first instance those where the sensation of effort is manifest; I will next examine those derived from it.

In lypemania, and especially in the forms called stupor and parphobus, one frequently observes the following hallucination, which is also met with in nightmare, and certain kinds of dreams. Some danger menaces you, an assassin, for instance; you are afraid, you fly; the assassin follows you; you fly more quickly, full of terrible distress; your feet scarcely touch the earth, you cross seas and mountains in an insensate course; the assassin follows you more rapidly still, and is about to overtake you. When this takes place in a dream, the climax of terror wakens you; but in madness, the awaking never comes, and the situation of the patient is horrible to behold. In both cases, the pulse is accelerated, the skin bathed with perspiration. The sensation of muscular activity is sometimes so strong, that the patient is fatigued as though he had actually run. The following is another remarkable example: You wish to fly, but an invincible force holds you back; you wish to defend yourself, but your arms remain without movement, in spite of the most energetic desire to do so; you wish to cry, but it is impossible. You are immovable as a living stone, and you waste yourself in superhuman efforts. Although there may have been no effective movement, the effort is sometimes so violent, as to cause the sensation of fatigue.

These hallucinations belong to the class which M. Baillarger has called psycho-sensorial. Others are purely psychical, according to the denomination of the same author.

In certain forms of mania, especially those which are complicated with lesion of the motive powers, the patients are not only unconscious of the paralysis which creeps on, but they experience sensations altogether different. Nothing is more common than to hear them say that their strength is doubled, that they can walk for whole days, carry on the extended arm enormous weights, or that they feel an unaccustomed vigour in all their members. This feeling of power and strength, coinciding sometimes with an advanced stage of paralysis which prevents the patient moving, offers a most striking contrast.

The inverse takes place with many lypemaniacs. They cannot move or walk; they have no muscles; with some there is even a sensation of fatigue in the members. The hallucination then becomes psycho-sensorial.

One might multiply examples. Thus erotic ideas awaken in madness, as in a dream, very distinct and detailed sensations of the corresponding action. But I do not propose to write a complete history, and I therefore pass to another order of hallucinations, which also has relation to the sense of musculation.

It is by the appreciation of muscular effort that we know whether an object is more or less heavy. Musculation corresponds with weight, as vision with light, calorition with heat. Thus all the subjective sensations of weight constitute so many muscular hallucinations. The sensation of weight may augment or diminish. In the first case, the patients discover in objects they handle an unaccustomed heaviness. Esquirol quotes the case of a lady, who rejected a *seringue* with horror because she believed it to be filled with mercury. But the object may be a part of the patient's own body, especially in the case of hypochondriacs. They sometimes cannot raise their arm; their head is so heavy that they imagine it full of metal, and that they can no longer sustain it on their shoulders. Some are convinced that it is entirely made of silver or lead.

Many insane persons have experienced the following sensation, which frequently occurs in dreams: They are on the brink of a well, or on the edge of a precipice, their foot slips from under them, and they fall. During the whole time that the descent lasts, one feels besides the anxiety, a sensation which can only be rendered by these words: one feels oneself falling. For the rest, it is the analogue of what passes when one really precipitates oneself from an elevation. This example appears to me to characterize the case where the hallucination, instead of limiting itself to the arm or the head, embraces the entire body.

When the sensation of weight diminishes or disappears, the patient believes himself to be so light, that he fears to be carried away by the slightest breath. Others feel as though effectually transported; they fly through the air; it was thus the witches went to their Sabbath. The following is what Jean Engelbrecht relates of himself. "On Thursday, at noon, I felt that death was near, and that it proceeded from the lower to the upper extremities. My body became stiff, and I lost all feeling in my feet, hands, and other parts. I could neither speak nor see. My mouth was paralysed; my eyes ceased to perceive the light. I distinctly heard the assistants say, 'Feel his legs, how cold they are; he will soon be dead.' I had no sense of touch; the hearing was also extinguished in its turn. *Then I was carried into space, with the speed of an arrow shot from a bow.*"—(Brierre de Boismont, *Hallucinations*, p. 265.)

In the ecstasy of mystics this phenomenon is very frequent. In the moment of transport they often feel themselves raised from the earth.

Saint Theresa once believed herself to be raised so violently, that she threw herself down with her face to the earth, making efforts not to fly away. Another saint, while praying on his knees, felt himself gently raised, and remained, as he thought, for some hours in the same position, several metres above the ground. The history of mystics is full of facts of this kind.

As we have seen, muscular hallucinations are frequent, and we shall easily find many examples of them; but I believe I ought not to enlarge the category in which I have placed them. I had thought in the first instance of adding the sensations described by Darwin, who thought he discovered in them a special sense of extension, but I reject these in order that I may keep strictly within the definition of the muscular sense which I gave at the beginning of this article.

I shall perhaps be reproached for having throughout these remarks compared the hallucinations of dreams with those of insanity. I have not done so without premeditation. Although from a clinical point of view these two states ought to be carefully distinguished, I consider that from a theoretical point of view whence my observations proceed, they may, and indeed, ought to be blended, for they are both characterized by an excess of subjectivity, temporary in one case, and more or less permanent in the other, but without any essential difference. When one falls asleep, in passing through the intermediate state so well recognised at the present day, the dream directly continues the hallucination, without the possibility of establishing the precise limit between the two; and reciprocally, certain dreams which have vividly impressed themselves, continue for some instants after waking, in such a manner as to constitute real hallucinations. Again, if we remark that in madness, hallucination often, as is proved by Englebrecht's case, occurs only when the senses fail to perceive the external world, we shall see here a new motive for comparing the states of dream and insanity with one another.—*Gazette Hebdomadaire*, 6*th Feb.*

